# A. V. M. A.

**Published:** 1901-07

**Authors:** 


### A. V. M. A.

THE following papers have been offered for the annual meeting of the American Veterinary Medical Association at Atlantic City, September 3, 4, and 5, 1901:

Dr. W. C. Fair, Cleveland, Ohio, "Lameness;" Dr. George R. White, Nashville, Tenn., "Municipal Meat-inspection, with Special Reference to that in Vogue in Nashville;" Dr. William McEachran, Windsor, Ont., "Distemper in the Dog;" Dr. D. P. Yonkerman, Kalamazoo, Mich., "The Treatment of Tuberculosis by the Salts of Copper;" Dr. G. W. Dunphy, Quincy, Mich., "Contagious Abortion of Cattle;" Dr. R. S. Huidekoper, Philadelphia, Pa., "Ethics of Veterinary Education;" Dr. G. E. Nesom, Clemson College, N. C., "Texas Fever in Native South Carolina Cattle;" Dr. V. A. Moore, Ithaca, N. Y., "Skin Disinfection and Wound Infection;" Dr. A. T. Peters, Lincoln, Neb., "Vaccination as a Preventive in Hog-cholera;" Dr. W. H. Dalrymple, "Anthrax and Preventive Inoculation in Louisiana;" Dr. L. A. Merillat, "The Value of the More Common Surgical Operations on the Horse;" Dr. Carl Gay, "The Attitude of the Farmer toward the Tuberculin Test."

Other papers, the subjects of which will be announced later, have been offered by Dr. C. H. Higgins, Montreal, Quebec; Dr. C. A. Cary, Auburn, Ala.; Dr. C. C. Lyford, Minneapolis, Minn.; Dr. E. M. Ranck, Philadelphia, Pa.

Dr. William G. Shaw, of Benson, Arizona, expects to attend the meeting at Atlantic City in September. In the month of May he inspected more than half as many cattle as were inspected at that point during the entire year of 1889.

